# High-risk surgical stage 1 endometrial cancer: analysis of treatment outcome

**DOI:** 10.1186/1748-717X-1-24

**Published:** 2006-08-03

**Authors:** Gustavo A Viani, Barbara F Patia, Antonio C Pellizzon, Marcel D De Melo, Paulo E Novaes, Ricardo C Fogaroli, Maria A Conte, Joao V Salvajoli

**Affiliations:** 1 Department of Radiation Oncology Hospital do Cancer, Sao Paulo, Brazil

## Abstract

**Purpose:**

To report the relapse and survival rates associated to treatment for patients with stage IC, grade 2 or grade 3 and IB grade 3 diseases considered high risk patients group for relapse.

**Materials and methods:**

From January 1993 to December 2003, 106 patients with endometrial cancer stage I were managed surgically in our institution. Based on data from the medical records, 106 patients with epithelial endometrial cancer met the following inclusion criteria: stage IC grade 2 or 3 and IB grade 3 with or without lymphovascular invasion. Staging was defined according to the FIGO surgical staging system. Postoperative adjuvant radiotherapy consisted of external beam pelvic radiation, vaginal brachytherapy alone or both. The median age was 65 years (range, 32–83 years), lymph node dissection was performed in 45 patients (42.5%) and 14 patients (13.2%) received vaginal brachytherapy only, and 92 (86.8%) received combined vaginal brachytherapy and external beam radiotherapy. The median dose of external beam radiotherapy administered to the pelvis was 4500 cGy (range 4000 – 5040). The median dose to vaginal surface was 2400 cGy (range 2000 – 3000). Predominant pathological stage and histological grade were IC (73.6%) and grade 3 (51.9%). The lymphovascular invasion was present in 33 patients (31.1%) and pathological stage IC grade 2 was most common (48. 1%) combination of risk factors in this group.

**Results:**

With a follow up median of 58.3 months (range 12.8 – 154), five year overall survival and event free survival were 78.5% and 72.4%, respectively. Locoregional control in five year was 92.4%. Prognostic factors related with survival in univariate analyses were: lymphadenectomy (p = 0.045), lymphovascular invasion (p = 0.047) and initial failure site (p < 0.0001). In multivariate analyses the initial failure in distant sites (p < 0.0001) was the only factor associated with poor survival. Acute and chronic gastrointestinal and genitourinary toxicity grades 3 were not observed.

**Conclusion:**

In conclusion, our results showed that the stage IC, grade 2, 3 and IB grade 3 endometrial cancer was associated with significantly increased risk of distant relapse and endometrial carcinoma-related death independently of salvage treatment modality.

## Background

Patients with stage I endometrial carcinoma, treated with total abdominal hysterectomy and bilateral salpingo-oophorectomy (TAH-BSO) and postoperative radiotherapy (RT) tailored to prognostic factors, have 5-year overall survival rates of 80% to 90%, 5-year cancer-specific survival of 90% to 95%, and locoregional recurrence rates of 4% to 8%. [1-8] However, the subgroup of patients with grade 3 tumors with deep (50% or more) myometrial invasion (stage IC, grade 3) has been reported to have a considerably higher risk of both locoregional and distant relapse. The Gynecological Oncology Group (GOG) staging study [9] showed the risk of microscopic pelvic node metastases for patients with clinical stage I endometrial carcinoma to be below 10%, except for those with outer 33% myometrial invasion, for whom the risk amounted to 18%. When designing the multicenter randomized Postoperative Radiation Therapy in Endometrial Carcinoma (PORTEC) trial for stage I endometrial carcinoma, [10] it was decided to exclude the subgroup of patients with grade 3 tumors with outer 50% myometrial invasion from random assignment in view of the reported higher relapse rates and because a survival benefit with pelvic RT had been suggested. In the Aalders et al. [1] study, a subgroup analysis in patients with deep myometrial invasion revealed that the rate of pelvic relapse was lower in the radiotherapy treated both grade 3 disease and deep invasion, a 10% decreased in the cancer death rate was seen with the addition of pelvic radiotherapy, and the pelvic relapse rate was lower, at 4.5% versus 20%. This series was done to report the relapse and survival rates for patients with stage IC, grade 2 or grade 3 and IB grade 3 disease with endometrial cancer considered high risk patients group for relapse. The secondary objective was to analyze the impact in survival of initial failure sites.

### Patients and methods

From January 1993 to December 2003, 250 patients with endometrial cancer stage I were managed surgically at Hospital do cancer (Sao Paulo, Brazil). Based on data from the medical records, 106 patients with epithelial endometrial cancer met the following inclusion criteria: (1) total hysterectomy and removal of existing adnexal structures with or without additional surgical staging procedures for endometrial cancer, (2) stage IC grade 2 or 3 and IB grade 3 with or without lymphovascular invasion and (3) no other malignancy diagnosed within 5 years before or after the diagnosis of endometrial cancer (except for carcinoma in situ or skin cancer other than melanoma). Staging was defined according to the International Federation of Gynecology and Obstetrics (FIGO-1992) surgical staging system. Postoperative adjuvant radiotherapy consisted of external beam pelvic radiation or vaginal brachytherapy or both. The interval time between surgery and radiotherapy treatment did not exceeded 4 weeks. The decision to deliver adjuvant radiotherapy depended predominantly on the assessment by gynecologic oncologists and radiation oncologists of the risks of local or regional both recurrence after pathologic evaluation of the surgical specimen. This decision usually was dictated by presence of grade 3 differentiation, nonendometrioid histological subtype, or deep myometrial invasion, or a combination of these pathologic features. The most part of patients were treated with pelvic "box technique" for a dose of 45 Gy in 5 weeks, with daily fractions of 1.8 Gy. Radiation was initiated no later than 8 weeks after surgery via cobalt60 teletherapy or linear accelerator with energy of 4 MeV or greater. Pelvic radiotherapy fields were standard with an upper border of L5-S1, while the inferior border was at the mid-portion of the obturator foramen. The lateral borders were set at 1 cm beyond the lateral margins of the bony pelvic wall at the widest plane of the pelvis. Lateral field borders were the posterior border of the S3 vertebral body and the anterior border of the symphysis pubis. Beam arrangement was 4-field. During irradiation patients were checked weekly with X-ray portal of control. Following pelvic radiation, the hypofractionated high dose rate vaginal vault brachytherapy was delivered postoperatively. All the patients completed the treatment in 8 weeks after the initial date of pelvic radiation. Under sterile conditions, a Foley catheter was placed. The simulation treatment planning process initially included placement of an auto-suture radio-opaque clip at the vaginal apex. The largest possible diameter of the vaginal cylinder was selected for treatment to decrease the vaginal mucosa dose and improve depth dose. Dummy sources were placed in the vaginal applicator during simulation and subsequently prior to each treatment for appropriate placement under fluoroscopic guidance. The position of the vaginal applicator was documented on ventrodorsal and lateral X-ray prior to every treatment. The intracavitary treatment radiation was delivered in four high dose rate applications with a median dose of 24 Gy (range 20–30 Gy). The point of prescription dose used in the vaginal brachytherapy was the vaginal surface or to 5 mm of the surface of applicator when vaginal brachytherapy was used alone. During irradiation patients were checked weekly for adverse treatment-related effects and post treatment during follow up time. Patients who experience acute gastrointestinal (frequency, diarrhea), genitourinary (frequency, dysuria), and chronic gastrointestinal (rectite, obstruction), genitourinary (hematuria, disury) classified according to RTOG criteria were registered.

### Follow-up studies

After treatment, patients were followed every 4 months for 2 years and then every 6 months for 1 year and then yearly. Pap smear and chest radiographs were performed yearly. History and physical examination, Karnofsky performance status (KPS), and documentation of major symptoms or adverse effects were performed at every visit. Blood work with count blood cells (CBC), platelets, liver enzyme tests, and creatinine as well as pap smears and chest X-rays were assessed when necessary. If sufficient follow-up information about survival and recurrence was not available in the clinical records, death certificates were obtained and letters were sent or telephone calls were made to patients and family physicians to obtain the information.

### Study endpoints

The primary purpose of study was report the locoregional failure, distant failure, event free survival, overall survival rates associated with treatment. Local failure was defined as recurrence with the area of the pelvis and vaginal cuff encompassed by the pelvic radiation field. Distance failure refers to recurrence in distance sites outside the treated area. The patients were followed after relapses and probability of death was calculated of according with primary relapse site and salvage treatment modality.

### Statistical methods

Patterns of recurrence were primary endpoints in this study, pelvic failure, locoregional failure, and distant metastases failure rates were estimated using the cumulative incidence method. Absolute survival and disease-free survival rates were estimated using the Kaplan-Meier method. The covariates examined in all cases for survival were: age, lymphovascular invasion, lymphadenectomy, initial site of relapse, pathological stage, histological grade, combination of pathological and histological grade, pelvic radiotherapy and salvage treatment. The time point for survival analyses was from the end date of the radiotherapy treatment. The log rank test was applied using the intent-to-treat analysis of all eligible patients to evaluate differences between regimens with respect to event free survival (EFS), Local control (LC) and overall Survival (OS). All factors with a P-value ≤ 0.05 at univariate analysis were entered into a multivariate analysis using the proportional hazards model (Cox Regression) with confidential interval of 99%. The hazard function survival (Kaplan Meier method) was used to estimate the probability to death of according with initial failure site in the period of follow up. The Fisher test was used to identify an association between endometrial cancer mortality and relapse, differences were considered statistically significant at P < 0.05.

### Characteristics of patients

The median age was 65 years (range, 32–83 years), lymph node dissection was performed in 45 patients (42.5%). Of the 106 patients who received adjuvant radiotherapy, 14 (13.2%) had vaginal brachytherapy only, and 92 (86.8%) had combined vaginal brachytherapy and external beam radiotherapy. Predominant pathological stage and histological grade were IC (73.6%) and grade 3 (51.9%). The lymphovascular invasion was present in 33 patients (31.1%) and subtype histology predominant was adenocarcinoma in 97 patients (91.5%). The pathological stage IC grade 2 was most common (48.1%) combination of risk factors in this group. The clinical and pathologic characteristics of these patients are summarized in Table 1.

**Table 1:** Characteristic of patients and treatment

**Age**	**Median (Y)**	**Range**
	65	32–83
**Pathological stage**	**Number**	**%**
Stage – IB	**28**	**26.4**
Stage – IC	**78**	**73.6**
**Histology grade**	**Number**	%
II	51	48.1
III	55	51.9
**Histology subtype**	**Number**	%
Adenocarcinoma	97	91.5
No adenocarcinoma	9	8.5
**Grade and Pathological stage**	**Number**	%
IB G3	**28**	26.4
IC G2	51	48.1
IC G3	27	25.5
**Radiotherapy type**	**Number**	%
RT + VB	**92**	86.8
VB	14	13.2
**Radiotherapy dose Gy**	**Median**	Range
RT	45	40 – 50.4
VB	24	20 – 30
**Lymphadenectomy**	**Number**	%
Present	61	57.5
Absent	45	42.5
**Lymphovascular invasion**	Number	%
Present	73	68.9
Absent	33	31.1

## Results

### Overall survival, event free survival and local control in five and ten year

**Figure 1:**
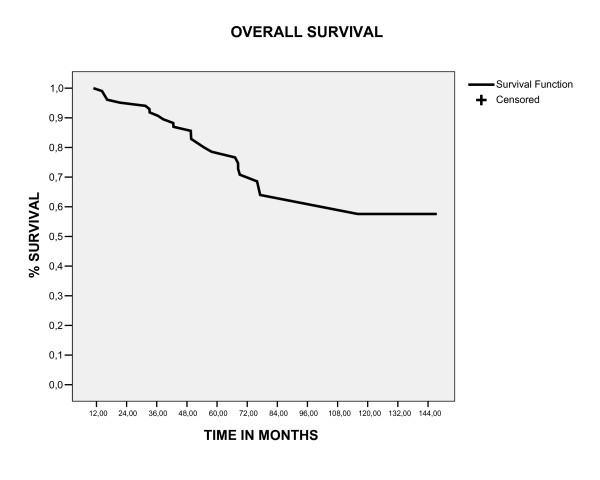
overall survival estimate by Kaplan Meier method.

**Figure 2:**
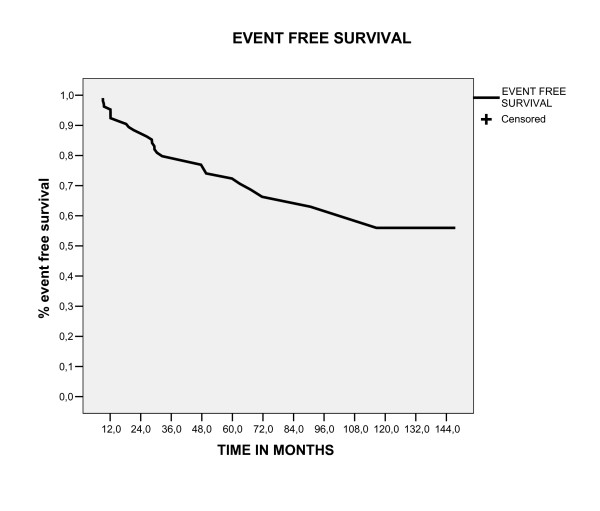
Event Free Survival estimate by Kaplan Meier Method.

**Figure 3:**
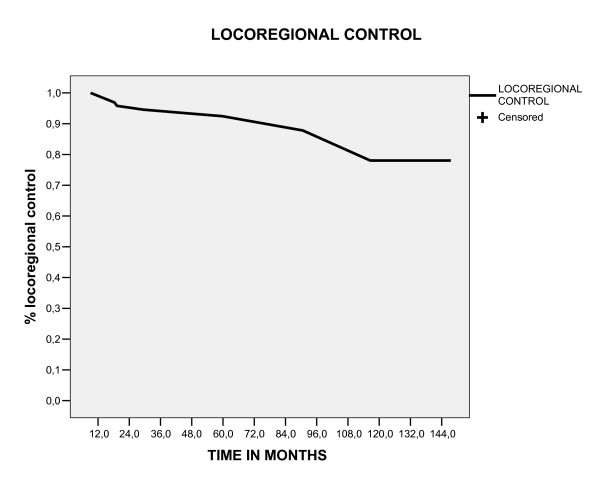
Loco regional control estimate by Kaplan Meier method.

With a follow up median of 58.3 months (range 12.8 – 154), the five and ten year overall survival and event free survival rates were 78.5% and 57.6%, 72.4% and 56%, respectively (figure 1, 2). The locoregional control rate in five and ten year was 92.4% and 78%(figure 3). The most frequent initial failure site was the distances site (73.3%), followed for pelvic recurrence in 16.7% of patients, as showed in table 3.

### Prognostic factors

**Figure 4:**
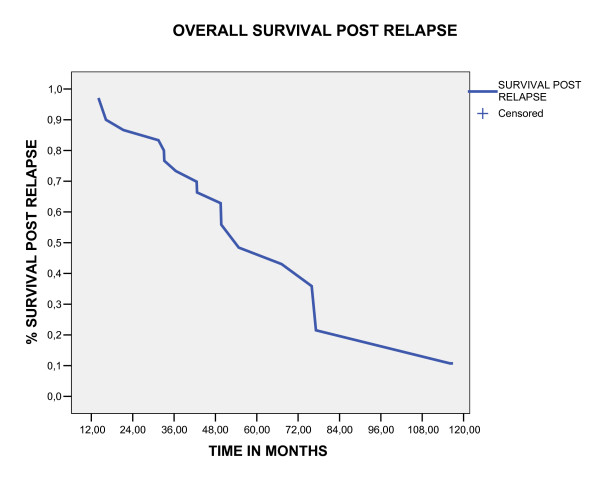
Overall survival post relapse estimate by Kaplan Meier method.

**Figure 5:**
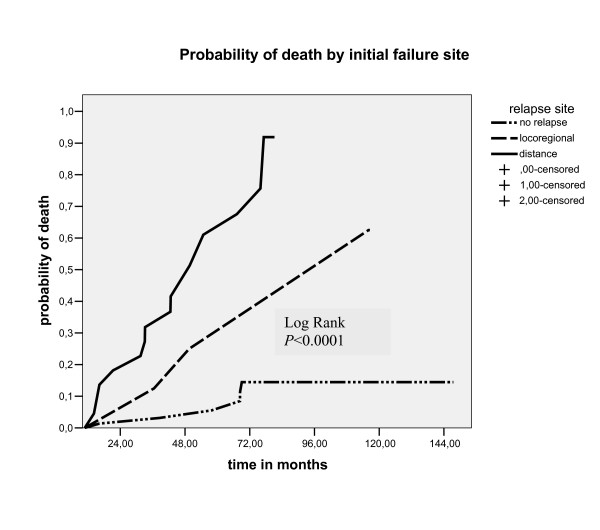
Probability of death by primary relapse site post rescue treatment.

In univariate analysis the factors associated with poor overall survival rates in five year were: presence of lymphovascular invasion space (p = 0.045), absence of lymphadenectomy (p = 0.047), distant site failure (p < 0.0001) and chemotherapy salvage treatment (p = 0.032), (table 2). In multivariate analyses the only factor that maintained associated with poor survival was the distant site failure (p < 0.0001), as showed in table xx. The initial failure in distant sites was associated with high probability of death by the five year (p < 0.0001, no relapse 5.5% vs loco regional relapse 25% vs distance sites 61%), as demonstrated in figure 5. Five year survival rate for the thirty patients who relapse was of 48.4%, with a median survival time of 54.7 months (CI 95% 29.7 – 79.7), as showed in figure 4. The group of patients who had relapses the endometrial cancer mortality rate was higher than in no relapse group (90% vs 16%, p = 0.002), as demonstrated in table 3.

**Table 2:** Univariate analysis to prognostic factors associates with OS in 5 years

***Five years OS***			
**Variable**	**Number**	**Event (%)**	**P value**
**Age**			
<60	55 – 51.8	12 – 80.4	***0.96***
>60	51 – 48.2	14 – 76.8	
**Pathological stage**	**Number %**	**Number %**	
Stage – IB	28 – 26.4	5 – 82	***0.53***
Stage – IC	78 – 73.6	25 – 7 7	
**Histology grade**	**Number %**	**Number **%	
II	51 – 48.1	12 – 84	***0.58***
III	55 – 51.9	14 – 73.7	
**Grade and Pathological stage**	**Number %**	**Number **%	
IB G3	28 – 26.4	5 – 82.3	***0.43***
IC G2	51 – 48.1	12 – 83.9	
IC G3	27 – 25.5	9 – 64.7	
**First failure site**	**Number %**	**Number **%	
No	76 – 71.6	6 – 94.5	
loco regional	8 – 4.7	3 – 75.5	***<0.0001***
Distance	22 – 20.7	17 – 39	
**Salvage treatment**	**Number %**	**Number %**	
Radiotherapy or surgery	13 – 12.2	5 – 77.4	***0.032***
chemotherapy	17 – 16.6	15 – 26.1	
**Lymphadenectomy**	**Number %**	Number %	
Present	61 – 37.5	6 – 88.7	***0.047***
Absent	45 – 42.5	20 – 71,6	
**Lymphovascular invasion**	Number %	Number %	
Present	73 – 68.9	13 – 71.2	***0.045***
Absent	33 – 31.1	13 – 82.2	

### Toxicity

According to RTOG criteria the genitourinary acute toxicity was: grade 1–20.7%, grade 2–5.6% and grade 3-0, respectively. The most frequent gastrointestinal acute toxicity was grade 1–21.6% follow by grade 2–7.5% and grade 3-0, as demonstrated in table 4. The genitourinary and gastrointestinal chronic toxicities grade 2 was 2.8% and 9.4%, respectively. No patients submitted to radiotherapy treatment had genitourinary or gastrointestinal chronic toxicity grade 3, as showed in table 4.

**Table 3:** Outcome by initial failure site and cause of death for patients with or without relapse.

**Outcome**	**Patients relapses**			
	**Yes (%)**	**No (%)**	**total (%)**	
***Initial failure site***	30	76		
				
***Vaginal***	3 (10)	0	3 (2.8)	
***Pelvic***	5 (17)	0	5 (4.7)	
***Distant***	22 (73)	0	22 (20.7)	
				
**Alive**	10 (7.9)	70 (92.1)	80 (75.8)	

**Outcome**	**Patients relapses**			
	**Yes**	**No**	**total**	p*

	30	76	106	
Death	20	6	26	
Endometrial Cancer	18	1	19	0.002
Others causes	2	5	7	

* Exact fisher test by association between relapse and cause of death

**Table 4:** Multivariate analyses of significant factors for survival (Cox Regression)

**VARIABLE**	**P**	HR*	**99% confidential interval**	
***Lymphadenectomy***				
*YES*	*0.34*	1(REF)**	*0.42*	*6.41*
*NO*		*1.66*		
***Lymphovascular invasion***				
*YES*	*0.48*	*1*(*REF*)	*0.51*	*5.18*
*NO*		*1.49*		
***Initial failure***				
*No or Local distance*	*<0.0001*	*1*(*REF*) *5.8*	*3.8*	*11.6*

## Discussion

All of the prospective studies made attempts to identify subgroups of patients at higher risk for recurrence. In the GOG study [11], a "high intermediate" group was defined by a combination of risk factors that included advanced age, lymphovascular invasion, outer-third invasion, and moderate to high tumor grade. As the first site of failure, the control arm of the low intermediate risk group (which comprised approximately two thirds of the patients) had an observed failure rate of 5%, while the higher risk group had a 13% risk for local-regional failure. The high intermediate risk patients were also at risk for failing distantly, with a 48-month observed distant failure rate of 19% in the control arm. The PORTEC trial [10] identified high-risk patients included patients older than 60, patients with stage IC, grade 1 or 2 tumors, and patients with stage IB, grade 3 tumors. This group of patients had a 5-year local-regional relapse rate of 19%, with the majority of relapses occurring in the vagina.

This analysis was done to investigate whether stage IC, grade 2 or grade 3 and IB grade 3 endometrial carcinoma should be considered a separate entity from the other prognostic subgroups of stage I endometrial carcinoma. The others objectives this study was report the relapses rate post radiotherapy and evaluate the impact of the initial failure site in patients survival. In the GOG 99 trial [11], patients with stage I to II endometrial cancer were randomly assigned after TAH-BSO with lymphadenectomy to receive pelvic RT or no further treatment. Interestingly, the results are strikingly similar to those obtained in the PORTEC study [10]: 88% 2-year relapse-free survival in the control group (17 locoregional recurrences in 200 patients) and 96% 2-year relapse-free survival in the RT group (three recurrences in 190 patients), with mainly vaginal recurrences in the control group.

There are limited data regarding outcome of *surgically staged *stage IC patients treated with observation alone for survival. Straughn et al. [12] reported the largest series (121 patients) treated with full surgical staging and no adjuvant radiotherapy; there was a 12% overall failure rate with 6% of patients failing locally; again, the vast majority of these local-regional failures were in the vagina and any benefit to survival was not showed.

Pelvic RT is generally recommended for grade 3 tumors with deep myometrial invasion. [13-16] In their review of radiation therapy for endometrial cancer, Koh et al[14] strongly recommend pelvic RT for surgically staged IC, grade 3 cancer, and suggest that RT be considered for grade 2 with outer 33% myometrial invasion. In a survey performed after the first results of GOG 99[11] had been reported, it was found that most GOG members (79%) would still recommended pelvic RT for stage IC, grade 3 disease.[13]

In our data pelvic lymphadenectomy was associated with better survival (p = 0.04) and was not related with any benefit to EFS and LC. This fact, in our study may be associated with reduced number of patients in the sample, compared to other studies, and the majority of patients was not submitted for pelvic lymphadenectomy (57. 5%).

The original GOG surgical-pathologic study found that lymphovascular invasion placed patients at high risk for lymph node metastases. Other investigators have confirmed this [17], and the risk for lymph node disease with lymphavascular invasion ranges from 20%–50% [18]. In our data, lymphavascular invasion was associated with poor overall survival rate in five year (81.1% vs. 52.8%, p = 0.043). Due to this, our data suggest that patients managed surgically without lymphadenectomy should be treated with pelvic radiotherapy in the presence of lymphovascular invasion, regardless of other risk factors.

**Figure 6:**
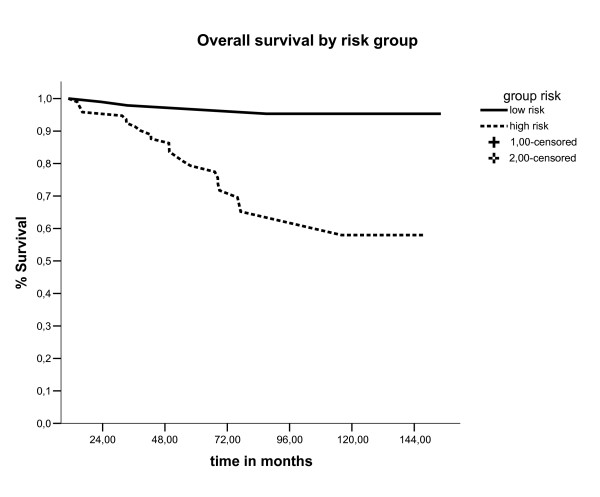
Overall survival by risk group (Kaplan Meier estimate).

The outcome patients in our study was comparable to reported relapse and survival rates for similar patients, [19] with overall survival, event free survival and locoregional control in five years of 78.5%,72.4% and 92.4%, respectively. Our data suggest that this particular patient subgroup (stage IC grade 2 or 3, stage IB grade3) should be considered a separate entity, because comparing this group of patients classified as high risk (106 patients) with the other group of our database, that was excluded of this analyses for being classified as low risk (144 patients), there was a significant difference in five year survival between these groups (97% vs 78.5%, p < 0.0001), as showed in figure 6. For this group of patients (high risk), in our analyses the most common of relapse site was the distant site (73.3%), followed by pelvic relapses (16.7%). Moreover, patients with distant relapse had an increased in the probability of death in five years (p < 0.0001) and salvage treatment with chemotherapy was associated with poor survival (P = 0.032), showing to be extremely difficult to salvage this patients. In this way, the disease-free and overall survival rates of high risk group endometrial cancers are strongly influenced by the increased distant relapse rates. This raises the question whether adjuvant chemotherapy would lower the risk of distant metastases and thus improve survival. Two randomized trials have been published that evaluated the efficacy of chemotherapy in the adjuvant setting. The first trial, using single-agent doxorubicin, did not show any benefit of adjuvant chemotherapy.[20] The first results of GOG 122, a randomized trial comparing whole-abdominal RT with combination doxorubicin plus cisplatin chemotherapy in advanced (stages III to IV) endometrial carcinoma, have been presented recently.[21] Combination chemotherapy was shown to improve both progression-free survival and overall survival rates (13% and 11% at 2 years, respectively) compared with whole-abdominal RT. Future trials should explore the optimal adjuvant therapy and the use of concurrent RT and chemotherapy.

In conclusion, our results show that the stage IC, grade 2, 3 and IB grade 3 endometrial cancer is associated with significantly increased risk of distant relapse and endometrial carcinoma-related death independently of salvage treatment modality. This group should be analyzed and treated separately from the other, more favorable stage I patients. Novel strategies should be investigated to increase the survival rates mainly for patients with high risk endometrial carcinoma.

**Table 5:** Acute and chronic toxicities according to RTOG.

**Acute toxicity**	**Radiotherapy treatment (%)**
***Genitourinary *(*disury*, *frequency*)**	
**RTOG**	
Grade 0	78 (73.5)
Grade 1	22 (20.7)
Grade 2	6 (5.6)
Grade 3	0
***Gastrointestinal *(*diarrheia*, *nausea*)**	
**RTOG**	
Grade 0	75 (70.7)
Grade 1	23 (21.6)
Grade 2	8 (7.5)
Grade 3	0
**Chronic toxicity**	**Radiotherapy treatment (%)**
***Genitourinary *(*disury*, *hematuria*)**	
**RTOG**	
Grade 0	99 (93.3)
Grade 1	4 (3.7)
Grade 2	3 (2.8)
Grade 3	0
***Gastrointestinal *(*obstruction*, *rectite*)**	
**RTOG**	
Grade 0	90 (85)
Grade 1	6 (5.6)
Grade 2	10 (9.4)
Grade 3	0
